# The Use of Machine Learning Algorithms in the Evaluation of the Effectiveness of Resynchronization Therapy

**DOI:** 10.3390/jcdd9010017

**Published:** 2022-01-10

**Authors:** Bartosz Krzowski, Jakub Rokicki, Renata Główczyńska, Nikola Fajkis-Zajączkowska, Katarzyna Barczewska, Mariusz Mąsior, Marcin Grabowski, Paweł Balsam

**Affiliations:** 1 1st Department of Cardiology, Medical University of Warsaw, 02-097 Warsaw, Poland; jakub.rokicki@wum.edu.pl (J.R.); renata.glowczynska@uckwum.pl (R.G.); marcin.grabowski@me.com (M.G.); pawel.balsam@me.com (P.B.); 2 Department of Medical Informatics and Telemedicine, Medical University of Warsaw, 00-582 Warsaw, Poland; 3 Cardiomatics, 31-339 Cracow, Poland; nikola.zajaczkowska@cardiomatics.com (N.F.-Z.); katarzyna.barczewska@cardiomatics.com (K.B.); mariusz.masior@cardiomatics.com (M.M.)

**Keywords:** artificial intelligence, heart failure, cardiac resynchronization therapy

## Abstract

Background: Cardiovascular disease remains the leading cause of death in the European Union and worldwide. Constant improvement in cardiac care is leading to an increased number of patients with heart failure, which is a challenging condition in terms of clinical management. Cardiac resynchronization therapy is becoming more popular because of its grounded position in guidelines and clinical practice. However, some patients do not respond to treatment as expected. One way of assessing cardiac resynchronization therapy is with ECG analysis. Artificial intelligence is increasing in terms of everyday usability due to the possibility of everyday workflow improvement and, as a result, shortens the time required for diagnosis. A special area of artificial intelligence is machine learning. AI algorithms learn on their own based on implemented data. The aim of this study was to evaluate using artificial intelligence algorithms for detecting inadequate resynchronization therapy. Methods: A total of 1241 ECG tracings were collected from 547 cardiac department patients. All ECG signals were analyzed by three independent cardiologists. Every signal event (QRS-complex) and rhythm was manually classified by the medical team and fully reviewed by additional cardiologists. The results were divided into two parts: 80% of the results were used to train the algorithm, and 20% were used for the test (Cardiomatics, Cracow, Poland). Results: The required level of detection sensitivity of effective cardiac resynchronization therapy stimulation was achieved: 99.2% with a precision of 92.4%. Conclusions: Artificial intelligence algorithms can be a useful tool in assessing the effectiveness of resynchronization therapy.

## 1. Introduction

Cardiovascular diseases remain the leading cause of death both in the European Union and worldwide [1]. Heart failure (HF) is a growing challenge due to the constantly increasing number of patients diagnosed with it. Moreover, the improvement of care in patients with myocardial infarction results in higher survival rates, but as a result, more patients suffer from HF. However, novel therapeutic options are simultaneously being developed and introduced into everyday clinical practice. Cardiac resynchronization therapy (CRT) stands as an option for patients with heart failure with reduced ejection fraction (HFrEF) for whom pharmacotherapy alone is insufficient to reduce symptoms and control cardiac function [2]. The aim of CRT is to restore physiological-like electrical heart activity, which is often altered in the course of the underlying disease. Clinical trials evaluating this method showed positive results in terms of relieving symptoms and, most importantly, improving the survival rate [3,4]. Unfortunately, not all patients benefit equally from CRT. In some cases, CRT can be classified as ineffective based on echocardiographic results, clinical evaluation, or ECG tracing. The reasons for a lack of improvement in a patient’s health after receiving a CRT device should be investigated on different grounds related to HF management. However, many patients benefit from optimizing the device’s settings; this is a time-consuming activity, and due to a shortage of resources (i.e., physicians’ time), it could potentially be useful to support it by novel methods. One example of such an approach is machine learning (ML), which is a subset of artificial intelligence (AI). ML uses algorithms that analyze, learn, and make decisions based on the data received. Liang et al. aimed to establish the predictors of response to CRT with ML. According to the presented results, the left bundle branch block, left ventricular end—systolic diameter, and history of percutaneous coronary intervention were the strongest predictors of CRT response [5]. In another trial, Howell et al. also analyzed a group of patients and their parameters with the objective of identifying early CRT response with ML. Interestingly, almost half of the 19 predictors were potentially modifiable. The model predicted CRT response with 70% accuracy, 70% sensitivity, and 70% specificity. However, it has been stated that further prospective trials are required [6]. A call for referral and optimization of care in patients with CRT has been recently made by three European cardiac societies [7]. This form of AI use seems to answer the current needs in terms of therapy optimization. Artificial intelligence is becoming more and more popular in selected diagnostic and treatment areas, but its role in evaluating CRT effectiveness is yet to be established. The aim of this project was to develop an AI algorithm based on ECG tracings and to evaluate its ability to detect ineffective CRT pacing. Applying this method in clinical practice could potentially result in increased efficiency and better care for patients.

## 2. Material and Methods

**Figure 1:**
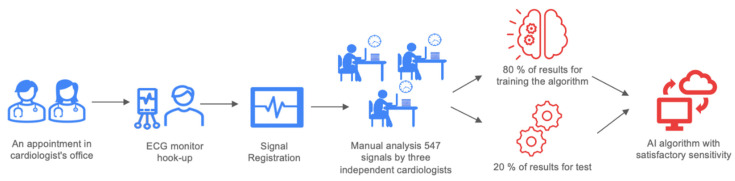
Project workflow.

**Figure 2:**
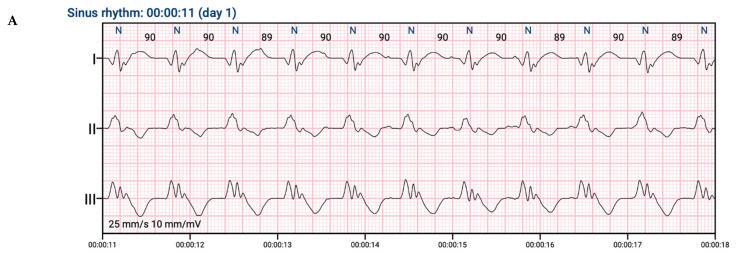

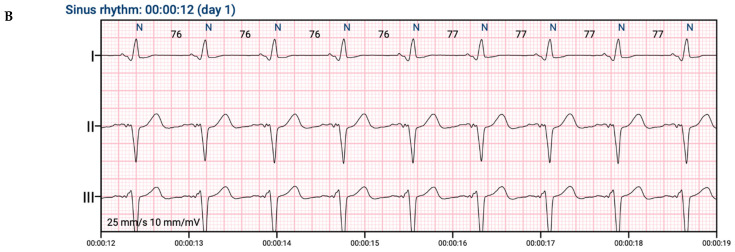
An example of an ECG in the same patient with CRT mode off (**A**) and on (**B**), which was used for AI algorithm training.

This study was an investigator-initiated, single-center, prospective observational trial. It was carried out in the First Department of Cardiology at the Medical University of Warsaw. All patients gave informed consent before any study-related procedures. This project was previously registered on www.clinicaltrials.gov (NCT04061434) (accessed on 19 August 2019). This study consisted of two independent groups of patients whose ECGs were collected using standard 30-s ECG recordings recorded with a Medea Kardio PCM (24-bit processing; Medea Sp. z o. o., Gliwice, Poland). All ECG signals were analyzed by three independent, experienced clinicians. Every tracing was manually classified by a physician and fully reviewed by senior cardiologists. The assessment of resynchronization was based on the QRS complex morphology. ECG patterns in CRT have been described before [8]. Moreover, a metanalysis concluded that QRS complex shortening after CRT implantation is associated with a favorable clinical and echocardiographic response [9]. The project workflow is presented in Figure 1. The study groups were as follows: recipients of CRT with pacemaker (CRT-P) or defibrillation function (CRT-D), patients after cardiac implantable electronic devices (CIED) such as a cardiac pacemaker, and patients with an implantable cardioverter defibrillator (ICD) with indications for periodic heart stimulation. Approval for all study groups was obtained from the institutional review board (AKBE/127/2018) according to the Declaration of Helsinki. In patients with an existing implanted device, the signal was recorded in pacing mode and standby mode (Figure 2), with the exception of pacing-dependent patients. Moreover, for the patients in the CRT-D/CRT-P group, the signal was registered with different configurations of stimulation (no stimulation, right ventricle pacing, left ventricle pacing, or biventricular pacing) and through stimulation of different regions of the left ventricle. 

As a result, each of the 1241 30-sec recordings from 541 patients was described by clinicians as an example of one of the four classes. The class assigned to the single record was assigned to each heartbeat within that record:

- Class 1: effective CRT stimulation;
- Class 2: ineffective CRT stimulation;
- Class 3: CRT patient’s own rhythm/heartbeat morphology;
- Class 4: control group.

The results were divided into two parts, and 80% of the results were used to train the algorithm. Based on those records, the algorithm was expected to obtain the ability to analyze CRT effectiveness based on ECGs. The remaining 20% of the ECGs were prepared for the test in order to establish the algorithm’s accuracy. All data were handled according to the General Data Protection Regulation (GDPR). The majority of the study group was men (*n* = 417; 76.37%) with a mean age of 68.58 ± 14.49 years.

Patients’ medical history was also acquired: comorbidities, qualification for device implantation, and other examinations at that time. In selected patients with typically good responses for CRT, the ECG signal was registered with the Holter method as well, which will be described elsewhere. The analyzing system for detecting arrhythmia consisted of a cloud-based software platform. The electrocardiographic signal captured and uploaded to the platform was analyzed using deep neural network algorithms. The software allowed the ECG standard report of the signal to be visualized and the acquired data to be analyzed in terms of CRT sufficiency. The platform is a medical device certified in the European Union (Certificate No. 60148244 and Certificate No. 60148245).

### 2.1. ECG Signal Processing

ECG recordings were processed according to the following algorithm:

1. R-detection: position of each heartbeat (understood as position of R-waves) in time was detected (by modified Pan–Tompkins algorithm) [10].
2. Heartbeat segmentation: recording was segmented into 2-sec windows containing each detected heartbeat centered on the R-wave.
3. Classification: a 2-sec window with labeled heartbeat was treated as an input to the classifier—deep convolutional neural network. Classifier was trained to distinguish 2 classes—effective and ineffective CRT pacing. As ineffective CRT pacing, all heartbeats from classes 2, 3, 4 were treated.

Classifier was trained in 3 different scenarios:

Intra-patient—train and test sets can contain examples from all recordings;
Inter-recording—train and test sets are separate in terms of recordings;
Inter-patient—train and test sets are separate in terms of patients.

### 2.2. Theory/Calculation

Standard statistical metrics were calculated:

Sensitivity = TP/P;
FPR = FP/N;
Precision = TP/(TP + FP).
Where:
TP—number of heartbeats effectively stimulated in CRT therapy, correctly classified;
FP—other heartbeats, falsely classified as effectively stimulated in CRT therapy;
FPR—false positive rate;
P—heartbeats effectively stimulated in CRT therapy;
N—other heartbeats.

**Figure 3:**
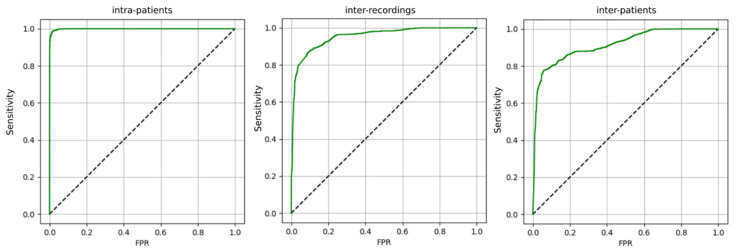
ROC curves for detection of the effectively stimulated heartbeats in CRT therapy, including different scenarios of model training.

ROC analysis was used to evaluate performance of the algorithm in the detection of effectively stimulated heartbeats for different decision thresholds. Figure 3 contains ROC curves for 3 different training scenarios.

The algorithm is able to successfully distinguish the effectively stimulated heartbeats in CRT therapy from the rest. From the point of view of the application in supporting the clinician’s work—it is beneficial to train the algorithm on the data from subsequent follow-up visits of various patients from the clinic (inter-recording vs. inter-patient). In order to achieve the highest efficiency, the algorithm should be adapted to the data from a particular patient (inter-patient vs. intra-patient)—e.g., by re-training the model on his recordings. In the future, in order to improve the results in the inter-patient or inter-recording scenario, adding additional parameters as input to the classifier can be considered, e.g., parameters from the echocardiography. However, due to previously accepted workflow and limited financing, further algorithm development was not possible at this stage. Further algorithm evolution and evaluation studies will be carried out in the future.

## 3. Results

The exact population characteristics are presented in Table 1. A total of 1241 recordings from 541 patients were collected. Because of poor recording quality, 2.56% of the ECG tracings were disqualified from the analysis. Three possible results were programmed for the AI algorithm: effective CRT, ineffective CRT, and baseline rhythm without any stimulation. The required level of sensitivity in the detection of effective CRT stimulation on the test set was achieved: 99.2% (with a precision of 92.4%).

Clinical verification of the algorithms was performed on 31 randomly selected Holter ECG records that well-reflected the representation of phenomena that the system has to deal with. The records were noted in great detail by the medical team, and the correctness of the annotation was checked with the accuracy of a single heartbeat. The error of the automatic analysis made by the system on the data set prepared in this way was determined at the level of single heartbeats, which amounted to 0.25%. 

## 4. Discussion and Conclusions

**Figure 4:**
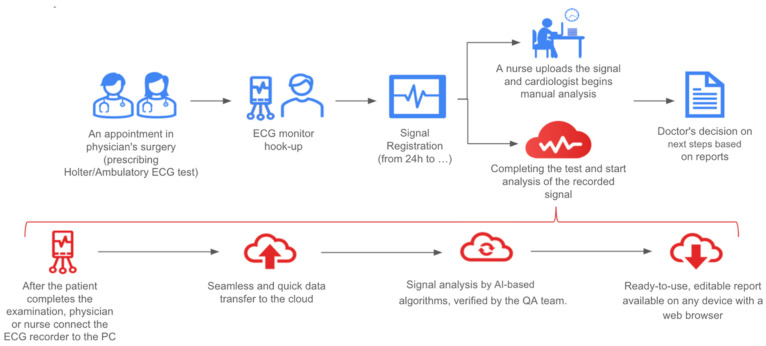
A potential workflow for AI use in everyday practice.

CRT is a widely used treatment option that can lead to improvements in a properly selected population. A positive change in quality of life and, most importantly, a reduction in mortality have been observed. Both the Comparison of Medical Therapy, Pacing and Defibrillation in Heart Failure (COMPANION) [5] and the Cardiac Resynchronisation—Heart Failure (CARE-HF) trials [4], which were the cornerstone of electrotherapy in HF patients, showed up to a 36% reduction in mortality, an effect size rarely seen in trials today. Moreover, these findings have been subsequently confirmed in real-world data registries and metanalyses [12,13]. On the other hand, some patients appear to not respond to CRT and show no clinical benefit from this intervention. According to the available data, up to one-third of patients may be classified as ‘non-responders’ [14]. Even though there are several criteria that help qualify patients as a ‘super-responder’, ‘responder’, ‘non-progressor’, ‘non-responder’, or ‘negative responder’, unique criteria for classification have not been adopted into everyday clinical practice so far. The reasons for a disappointing clinical effect could include device programming, HF pharmacology, or comorbidity management, and should be meticulously investigated by the HF center team [15]. In some cases, a response can be assessed based on an ECG. There are several parameters described in the Methods section that need to be evaluated and analyzed. There is no single cut-off point below which CRT is regarded as ineffective, but a higher percentage of stimulation is desirable [16]. Obviously, longer observation (i.e., 24-h Holter ECG monitoring) is beneficial and can yield more information on the mechanism behind a lack of response to CRT. Unfortunately, more data means more valuable physicians’ time is needed to assess it. Therefore, this process needs to be augmented. The constant progress of telemedicine and new methods result in new alternatives for practicing clinicians. Moreover, telemedicine is recommended by international cardiac societies [17,18]. AI seems to be a powerful tool, yet it is currently underused. Doctors across the globe are learning how to use it with good clinical effects for their patients. The aim of AI is to provide a set of tools and solutions to help clinicians. It can also provide patients with faster and more personalized care [19]. One example of AI use was described by Maille et al., who conducted a prospective observational study in which QTc duration was assessed by AI (Cardiologs^®^, Paris, France), comparing smartwatch single-lead ECGs with those measured on 12-lead ECGs in patients with early-stage COVID-19 being treated with a hydroxychloroquine–azithromycin regimen. No significant differences were observed between the two methods, but a need for further evaluation was highlighted [20]. Another example was presented by Smith et al., who described the results of a deep neural network by Cardiologs for full 12-lead ECG analysis, including rhythm, QRS, and ST-T-U waves, in comparison with Mortara/Veritas^®^ in emergency department ECGs. The algorithm was more accurate and specific in identifying previously specified changes. Additionally, it had a significantly higher rate of accurate ECG interpretation, with similar sensitivity and higher positive predictive value [21]. An ECG classification for convolutional neural networks has been proposed, and optimistic results were obtained in detecting ventricular ectopic beats and supraventricular ectopic beats in comparison with the state-of-the-art methods [14]. The use of novel methods, including AI, in treating and addressing possible pitfalls in heart rhythm disturbances is gaining more and more attention and is supported by global leaders in the field [22]. To the best of the authors’ knowledge, no AI-augmented solution for CRT optimization has been proposed so far. It should also be pointed out that device implantation entails several risks, and a risk–benefit analysis should always be conducted. A theoretical case of a patient who did not respond to CRT and suffered from infective endocarditis related to an implanted device is undesirable. Therefore, efforts should be made to enable the largest possible group of patients to benefit from CRT. The AI algorithm described in this paper showed very positive results and its clinical application could be valuable in many aspects. A possible workflow is presented in Figure 4. Physicians’ valuable time can be better managed and CRT effectiveness more quickly analyzed. As a result, CRT settings can be optimized, possibly allowing more patients to benefit from improved prognoses. A clinician could provide more patients with professional care instead of analyzing long ECG tracing. Data management and safety can be regarded as a possible threat, but proper information safety was a priority for Cardiomatics. 

In summary, AI augmentation bears minimal risk with a huge potential for clinical help. A saying is often repeated at conferences: ‘AI will not replace physicians now, but physicians using AI will replace ones who refuse to use it.’ This seems to be closely related to the issue at hand. Clinical evaluation is needed for further recommendations, but the presented results are promising.

## Figures and Tables

**Table 1:** Patient characteristics.

Characteristic		Overall Population
Male		417 (76.37)
Mean Age		68.58 ± 14.48
Heart failure symptoms [11]	0	148 (27.11)
	I	15 (2.75)
	II	193 (35.35)
	III	142 (26.01)
	IV	48 (8.79)
Myocardial Infarction History		200 (36.63)
Atrial fibrillation	Paroxysmal	98 (17.95)
	Persistent	24 (4.4)
	Permanent	116 (21.25)
Treatment		
Oral anticoagulation		301 (55.13)
Beta-blocker		483 (88.46)
ACE-inhibitor		302 (55.31)
Angiotensin receptor blocker		115 (21.06)
Antiarrhythmic drugs		149 (27.29)
MRA		256 (46.89)
Diuretic-loop		316 (57.88)
Diuretic-thiazide		57 (10.44)
Statins		361 (66.12)

For continuous variables, values are mean ± standard deviation; for categorical variables, *n* (%) is shown.

## Data Availability

The data will be shared upon reasonable request to the corrensponding author.

## Abbreviations

ECG—electrocardiogram; HF—heart failure; CRT—cardiac resynchronization therapy; HFrEF—heart failure with reduced ejection fraction; AI—artificial intelligence; CRT-P—CRT with pacemaker; CRT-D—CRT with defibrillation function; CIED—cardiac implantable electronic devices; ICD—implantable cardioverter defibrillator; MLP—multilayer perceptron; CNN—convolutional deep neural networks; GDPR—General Data Protection Regulation.
